# Protein Phosphatase 2A Interacts with the Na^+^,K^+^-ATPase and Modulates Its Trafficking by Inhibition of Its Association with Arrestin

**DOI:** 10.1371/journal.pone.0029269

**Published:** 2011-12-29

**Authors:** Toru Kimura, WonSun Han, Philipp Pagel, Angus C. Nairn, Michael J. Caplan

**Affiliations:** 1 Department of Pharmacology and Toxicology, Kyorin University School of Medicine, Mitaka, Tokyo, Japan; 2 Departments of Cellular & Molecular Physiology and; 3 Psychiatry, Yale University School of Medicine New Haven, Connecticut, United States of America; Cornell University, United States of America

## Abstract

**Background:**

The P-type ATPase family constitutes a collection of ion pumps that form phosphorylated intermediates during ion transport. One of the best known members of this family is the Na^+^,K^+^-ATPase. The catalytic subunit of the Na^+^,K^+^-ATPase includes several functional domains that determine its enzymatic and trafficking properties.

**Methodology/Principal Findings:**

Using the yeast two-hybrid system we found that protein phosphatase 2A (PP2A) catalytic C-subunit is a specific Na^+^,K^+^-ATPase interacting protein. PP-2A C-subunit interacted with the Na^+^,K^+^-ATPase, but not with the homologous sequences of the H^+^,K^+^-ATPase. We confirmed that the Na^+^,K^+^-ATPase interacts with a complex of A- and C-subunits in native rat kidney. Arrestins and G-protein coupled receptor kinases (GRKs) are important regulators of G-protein coupled receptor (GPCR) signaling, and they also regulate Na^+^,K^+^-ATPase trafficking through direct association. PP2A inhibits association between the Na^+^,K^+^-ATPase and arrestin, and diminishes the effect of arrestin on Na^+^,K^+^-ATPase trafficking. GRK phosphorylates the Na^+^,K^+^-ATPase and PP2A can at least partially reverse this phosphorylation.

**Conclusions/Significance:**

Taken together, these data demonstrate that the sodium pump belongs to a growing list of ion transport proteins that are regulated through direct interactions with the catalytic subunit of a protein phosphatase.

## Introduction

Ion transporting pumps generate ion gradients across membranes, and these gradients are essential for cellular homeostasis. The P-type ATPase family includes the Na^+^,K^+^-ATPase, Ca^2+^-ATPase, H^+^,K^+^-ATPase, heavy metal transporting ATPases and yeast plasma membrane H^+^-ATPase. The catalytic subunits of these ATPases have similar structures and functions [1], [2]. However, their intracellular distributions. the ions transported, and their regulation are quite different. Clearly, there must be specific function-determining domains in each ATPase that define their individual properties. Furthermore, each ATPase is likely to interact with specific proteins that help to determine their individual trafficking and regulation properties.

The Na^+^,K^+^-ATPase, or sodium pump, is expressed ubiquitously in virtually all tissues and plays a key role in the maintenance of intracellular electrolyte homeostasis [3]. The Na^+^,K^+^-ATPase consists of two subunits. The catalytic α-subunit contains 10 transmembrane domains and within its structure reside the sites for ion recognition, ATP and inhibitor binding, and protein kinase A (PKA) and protein kinase C (PKC) phosphorylation [4], [5], [6]. The glycoprotein β-subunit has a single transmembrane domain. It is also essential for the functional expression of Na^+^,K^+^-ATPase and is involved in the pump's structural maturation [7]. In specific tissues, the Na^+^,K^+^-ATPase can associate with a γ-subunit that alters its catalytic properties [8], [9], [10], [11], [12], [13], [14], [15]. Structural and biochemical studies demonstrate that the domain from TM4 to TM5 of the Na^+^,K^+^-ATPase α-subunit forms a large intracellular loop that is important for the pump's catalytic cycle, because it contains both the ATP binding site and the catalytic phosphorylation site [4], [5], [16]. ATP hydrolysis catalyzed by this domain provides the energy that the pump invests in Na^+^ and K^+^ transport.

We have conducted yeast two hybrid screening to look for proteins that interact with the Na^+^,K^+^-ATPase [17]. The domain from TM4 to TM5 of the Na^+^,K^+^-ATPase α-subunit and a human kidney cDNA library were used as the bait and prey, respectively. We found protein phosphatase 2A (PP2A) C-subunit to be one of the candidate partner proteins. Recently, Lecuona et al showed that the first 90 amino acids of the Na^+^,K^+^-ATPase α-subunit also directly interacted with PP2A C-subunit [18].

PP2A is one of four major cytoplasmic serine/threonine phosphatases and accounts for a large portion of the total phosphatase activity in many cells. The core enzyme of PP2A comprises a 36 kDa catalytic (C−) subunit that is always associated with a 65 kDa scaffolding subunit, called A or PR65, which modulates its enzymatic properties [19]. Distinct classes of regulatory (B−) subunits can bind to A and C heterodimers to form a wide variety of heterotrimeric complexes [20]. ABC heterotrimers are the most prevalent forms of PP2A in vivo [21].

It has been demonstrated that trafficking and signaling of G-protein coupled receptors (GPCRs) are regulated differently by both arrestins [22], [23], [24], [25] and spinophilin [25], [26], [27] through direct association. These associations depend on the phosphorylation of GPCRs by G-protein coupled receptor kinases (GRKs). We have shown that the Na^+^,K^+^-ATPase α-subunit is phosphorylated by GRKs, associates with both arrestins and spinophilin, and that these associations may modulate trafficking of the Na^+^,K^+^-ATPase [28]. Since PP2A is one of the major cellular phosphatases, we hypothesized that it may regulate GRK phosphorylation of the Na^+^,K^+^-ATPase and its association with arrestin. In addition, the Na^+^,K^+^-ATPase is regulated both by PKA and PKC phosphorylation and by dephosphorylation through the action of phosphatases [6], [29], [30], [31], [32], [33], [34], [35]. Here, we show that PP2A C-subunit associates directly with the Na^+^,K^+^-ATPase both in vitro and in vivo, and that PP2A expression can regulate the intracellular trafficking of the Na^+^,K^+^-ATPase.

## Results

### Localization of Na^+^,K^+^-ATPase and PP2A in rat kidney

**Figure 1:**
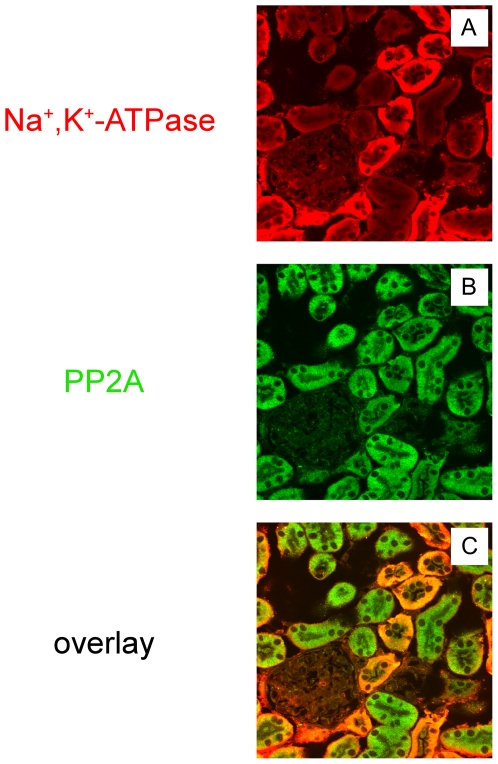
Immunolocalization of Na^+^,K^+^-ATPase and PP2A in situ. Mouse kidney sections were stained with the Na^+^,K^+^-ATPase antibody (A) and PP2A C-subunit (PP2A) antibody (B). Merged images are shown in C. (×40 magnification) The Na^+^,K^+^-ATPase and PP2A partially co-localize within the basolateral infoldings of proximal tubule epithelial cells. Typical results from one of three experiments are shown.

We have previously found through a yeast two hybrid screen and GST pull down assay that the PP2A C-subunit is one of the candidate proteins that interact with a cytoplasmic portion of the Na^+^,K^+^-ATPase α-subunit [17]. To confirm that Na^+^,K^+^-ATPase and PP2A localize to the same subcellular structures in a physiologically relevant tissue, immunocytochemistry was performed on sections prepared from rat kidney. Rat kidney sections were labeled with an anti-Na^+^,K^+^-ATPase antibody and with an antibody directed against the PP2A C-subunit (Fig. 1). Na^+^,K^+^-ATPase was expressed at the basolateral membrane of renal tubule epithelial cells. Na^+^,K^+^-ATPase staining was not detected in the glomerulus [36], [37]. As expected, expression of the Na^+^,K^+^-ATPase was higher in distal tubules than in proximal tubules (Fig. 1 A). PP2A was present in proximal tubule as well as in distal tubules (Fig. 1 B). The Na^+^,K^+^-ATPase and PP2A were partially co-localized along the basolateral infoldings of epithelial cells in the proximal tubules (Fig. 1 C). The same immunostaining patterns were obtained when rat kidney sections were examined with an anti-PP2A A-subunit antibody (data not shown).

### Immunoprecipitation from kidney tissue

**Figure 2:**
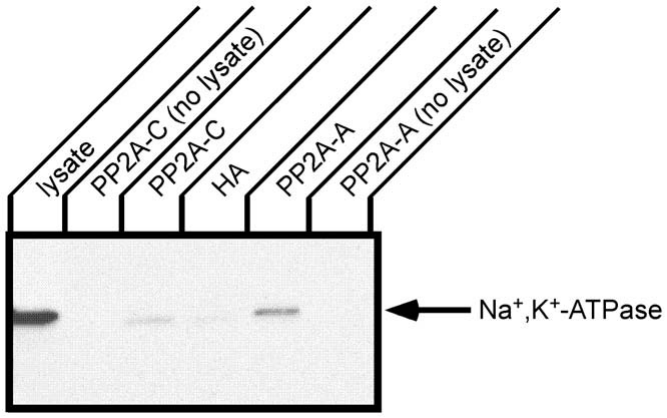
Immunoprecipitation of Na^+^,K^+^-ATPase and PP2A from rat kidney. Rat kidney lysate was incubated with antibodies directed against the PP2A C-subunit, the PP2A A-subunit or the HA epitope (control) followed by protein A beads. As an additional control for the fact that the Na,K-ATPase α-subunit migrates in SDS-PAGE in close proximity to the band corresponding to IgG heavy chain dimers, the antibodies directed against the PP2A A-subunit or C-subunit were incubated with lysis buffer without the addition of tissue lysate. Immune complexes were separated by SDS-PAGE and Western blotting was performed with biotinylated anti Na^+^,K^+^-ATPase antibody, 6H. The Na^+^,K^+^-ATPase was co-precipitated with both the C- and A-subunits of PP2A. Typical results from one of three experiments are shown.

Immunoprecipitation was performed from kidney tissue to determine whether the Na^+^,K^+^-ATPase associates with PP2A *in situ* (Fig. 2). Immunoprecipitations were performed with PP2A A- or C-subunit antibodies and an antibody directed against the HA epitope as a negative control. The Na^+^,K^+^-ATPase α-subunit that associated and co-precipitated with PP2A was detected by Western blotting with biotinylated anti-Na^+^,K^+^-ATPase α-subunit antibody. The biotinylated Na^+^,K^+^-ATPase α-subunit antibody was employed to avoid the need for a secondary antibody that might also detect the antibodies that were used for immunoprecipitation. When a control antibody (anti-HA epitope tag) was used for immunoprecipitation, an extremely faint band co-migrating with the Na^+^,K^+^-ATPase α-subunit was detected. In contrast, both the anti-PP2A A- and C-subunit antibodies clearly co-precipitated readily detectable quantities of the Na^+^,K^+^-ATPase α-subunit. The amount of the α-subunit pulled down was greater with the PP2A A-subunit antibody as compared to when the C-subunit antibody was employed. This difference may reflect differing accessibility of the relevant antigenic site to the PP2A A- or C-subunit antibodies in the Na^+^,K^+^-ATPase/PP2A complex *in situ*. Similarly, the PP2A A- and C-subunit antibodies may possess differing affinities for their respective antigens. In either case, this result supports the conclusion that the Na^+^,K^+^-ATPase associates with PP2A *in situ*.

### Characterization of the interaction of Na^+^,K^+^-ATPase α-subunit and PP2A C- and A-subunits

**Figure 3:**
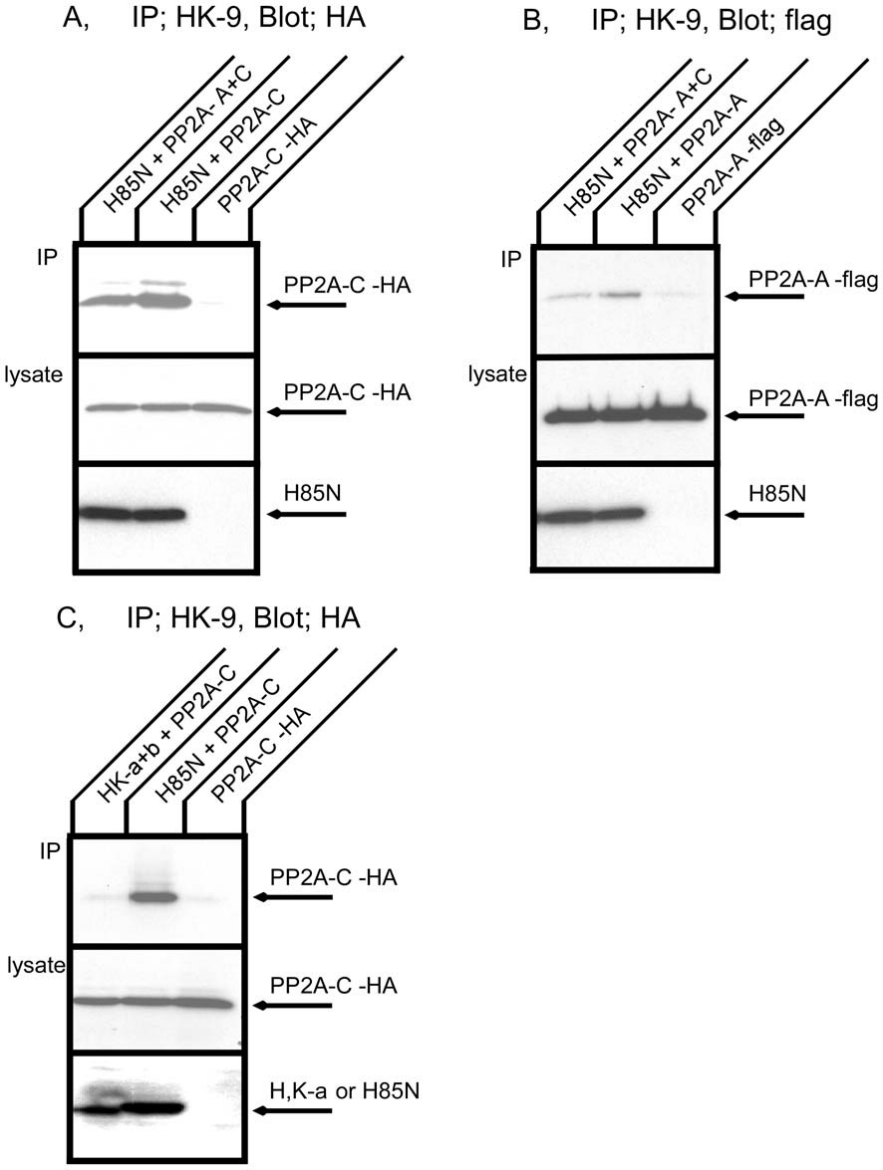
Co-immunoprecipitation of PP2A and the Na^+^,K^+^-ATPase or the H^+^,K^+^-ATPase expressed in COS cells. A. COS cells were transfected with HA tagged PP2A C-subunit alone, H85N plus HA tagged PP2A C-subunit, or H85N, flag tagged PP2A A- and HA tagged C-subunits. Immunoprecipitation was performed with HK9 antibody directed against the N terminus of H85N, and PP2A C-subunit was detected by Western blotting with anti-HA antibody. The quantity of H85N present in the cell lysates is detected by blotting with HK9 in the bottom panel. B. COS cells were transfected with flag tagged PP2A A-subunit alone, H85N plus flag tagged PP2A A-subunit, or H85N, flag tagged PP2A A- and HA tagged C-subunits. Immunoprecipitation was performed with HK9 antibody and PP2A A-subunit was detected by Western blotting with anti-flag antibody. The quantity of H85N present in the cell lysates is detected by blotting with HK9 in the bottom panel. Both the A- and C-subunits co-precipitated specifically with the Na^+^,K^+^-ATPase α-subunit. C. COS cells were transfected with HA tagged PP2A C-subunit alone, H^+^,K^+^-ATPase α- and β-subunits plus HA tagged PP2A C-subunit, or H85N plus HA tagged PP2A C-subunit. Immunoprecipitation was performed with HK9 antibody and PP2A C-subunit was detected by Western blotting with anti-HA antibody. The quantities of H85N and H^+^,K^+^-ATPase α-subunit present in the cell lysates are detected by blotting with HK9 in the bottom panel. The PP2A C-subunit was not immunoprecipitated with the H^+^,K^+^-ATPase. Typical results from one of five experiments are shown.

We investigated the dependence of the interaction between the Na^+^,K^+^-ATPase and PP2A upon the expression of the PP2A A- or C-subunits. For these experiments, COS cells were co-transfected with cDNAs encoding HA- or flag-tagged PP2A subunits as well as with a cDNA encoding the H85N chimera α-subunit construct. H85N is a chimera in which the first 85 residues of the Na^+^,K^+^-ATPase α-subunit are replaced by those of the gastric H^+^,K^+^-ATPase. This chimera manifests functional properties identical to those of the Na^+^,K^+^-ATPase and is recognized by the HK9 antibody directed against the N-terminus of the H^+^,K^+^-ATPase α-subunit [38]. Fig. 3A shows Western blot patterns of transfected COS cell lysates subjected to immunoprecipitation with the HK9 antibody and then detected with the anti-HA antibody, which recognizes the exogenous PP2A C-subunit. As expected, when cells were transfected only with HA-C-subunit, very little PP2A C-subunit was observed in the HK9 immunoprecipitate. In contrast, we found that PP2A C-subunit was immunoprecipitated when H85N was co-expressed with PP2A C-subunit. PP2A C-subunit was also detected in HK9 immunoprecipitates when cells were transfected with H85N and both the PP2A A- and C-subunits. PP2A A-subunit had no apparent enhancing or inhibitory effect on the interaction between the PP2A C-subunit and the Na^+^,K^+^-ATPase α-subunit. Fig. 3B demonstrates co-immunoprecipitation of H85N and flag-A-subunit. Once again, very little PP2A A-subunit was detected in HK9 immunoprecipitation when cells were transfected with PP2A A-subunit alone. PP2A A-subunit was immunoprecipitated with H85N both in the absence and presence of exogenous PP2A C-subunit. Interaction between the PP2A A-subunit and H85N was reduced somewhat in the presence of excess PP2A C-subunit. These results indicate that the Na^+^,K^+^-ATPase α-subunit forms a complex with both of the exogenously expressed PP2A A- and C-subunits.

### Lack of interaction between PP2A and a homologous P-type ATPase

Gastric H^+^,K^+^-ATPase is a member of the P-type ATPase family and a very close relative of the Na^+^,K^+^-ATPase. The H^+^,K^+^-ATPase is composed of two subunits and has the same topology as the Na^+^,K^+^-ATPase. H^+^,K^+^-ATPase α- and β-subunits and HA tagged PP2A C-subunit were transiently co-expressed in COS cells and immunoprecipitation was performed with HK9 antibody (Fig. 3C). In contrast to the results obtained with H85N, the PP2A C-subunit was not co-precipitated with the H^+^,K^+^-ATPase. We confirmed that the H^+^,K^+^-ATPase β-subunit was precipitated with the H^+^,K^+^-ATPase α-subunit under these conditions (data not shown). This result indicates that there is specificity in the binding of PP2A to P-type ATPase family members.

### GST pull down using in vitro translated products

**Figure 4:**
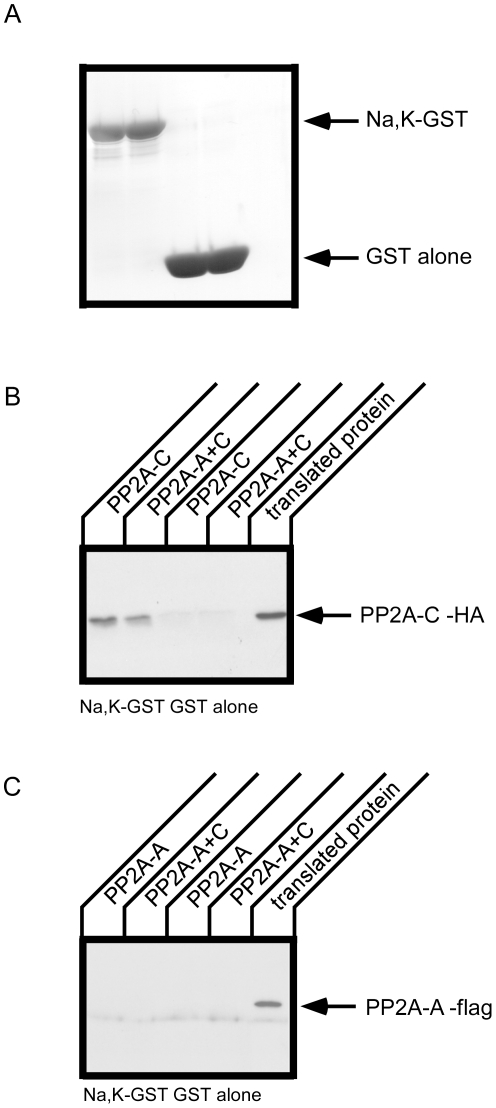
In vitro translation of PP2A and pull down with a GST construct incorporating the large cytoplasmic loop of the Na^+^,K^+^-ATPase α-subunit. PP2A A (flag tagged)- and C (HA tagged)- subunit proteins were prepared by in vitro translation and GST pull down was performed with GST alone or GST-Na^+^,K^+^-ATPase large cytoplasmic loop (Na,K-GST). A. GST proteins used for pull down were detected by coomassie brilliant blue (CBB) staining. PP2A C-subunit and A-subunit were detected by Western blot with anti-HA (B) and anti-flag (C) antibodies, respectively. The PP2A C-subunit, but not the A-subunit, specifically co-precipitate with the GST-Na^+^,K^+^-ATPase. Typical results from one of three experiments are shown.

In the results shown in Fig. 3, it is possible that the flag-A-subunit was not bound directly to the Na^+^,K^+^-ATPase α-subunit, but was instead bound through an interaction with endogenously expressed PP2A C-subunit. To examine this issue we performed a GST pull down assay using *in vitro* translated PP2A subunit proteins (Fig. 4). The PP2A A- or C-subunit was prepared separately by in vitro translation and utilized in GST pull downs with the Na^+^,K^+^-ATPase loop. The Na^+^,K^+^-ATPase loop pulled down PP2A C-subunit in the absence and presence of the PP2A A-subunit. The PP2A A-subunit, however, was not pulled down with Na^+^,K^+^-ATPase loop. In vitro translated PP2A A- and C-subunits did not appear to form a complex with one another in the translation mix, as evidenced by the fact that PP2A A-subunit was not pulled down even in the presence of PP2A C-subunit. These results suggest that the PP2A C-subunit is necessary for the association of PP2A and the Na^+^,K^+^-ATPase large cytoplasmic loop. As demonstrated below, the PP2A A-subunit appears to bind directly to a different cytoplasmic domain of the Na^+^,K^+^-ATPase.

### Sites of interaction between the large cytoplasmic loop of the Na^+^,K^+^-ATPase and the PP2A-C subunit

**Figure 5:**
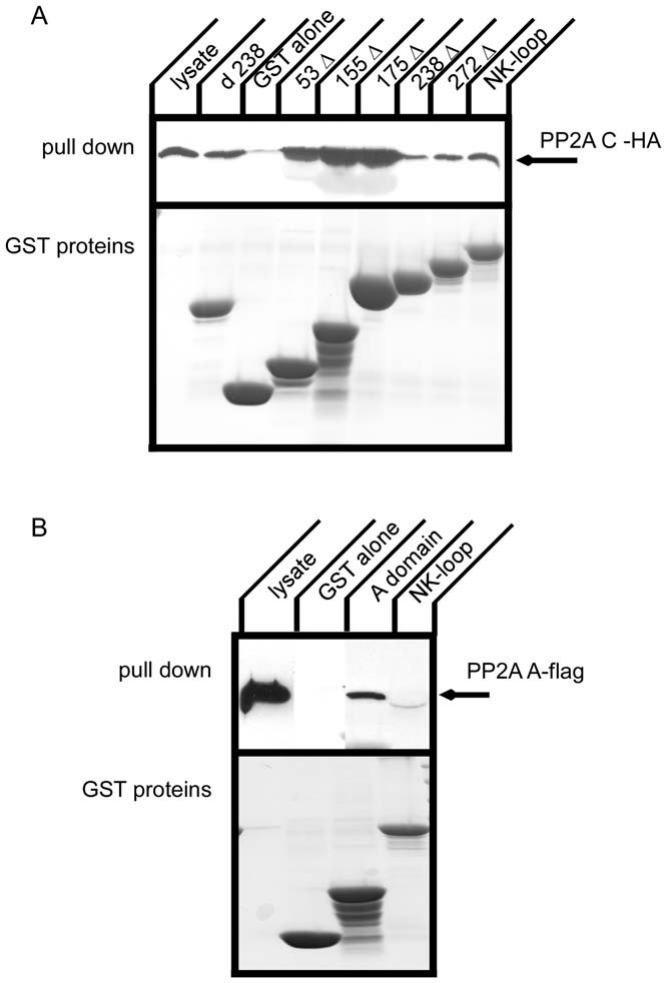
Deletion constructs of the large cytoplasmic loop of the Na^+^,K^+^-ATPase α-subunit and GST pull down of PP2A with GST constructs. A. HA tagged PP2A C-subunit was expressed in COS cells and cell lysates were incubated with GST fusion proteins. PP2A C-subunit was detected by Western blot with anti-HA antibody (upper panel) and GST fusion proteins were detected by CBB staining. Not only N-terminal segments but also the C-terminal half of the large cytoplasmic loop binds to PP2A C-subunit. Typical results from one of four experiments are shown. B. Flag tagged PP2A A-subunit was expressed in COS cells and cell lysates were incubated with GST fusion proteins. PP2A A-subunit was detected by Western blot with anti-flag antibody (upper panel) and GST fusion proteins were detected by CBB staining. The PP2A A-subunit co-precipitated with the A-domain of the Na^+^,K^+^-ATPase α-subunit. Typical results from one of three experiments are shown.

To narrow down the region of the Na^+^,K^+^-ATPase α-subunit large cytoplasmic loop (Na^+^,K^+^-loop) that interacts with the PP2A C-subunit, deletion constructs were employed in a GST pull down. The Na^+^,K^+^-loop is constituted of 415 amino acids. We generated GST fusion constructs in which portions of the Na^+^,K^+^-loop were deleted stepwise from the C-terminus [28] . A deletion from the N-terminal side of the cytoplasmic loop was also generated. Resultant GST fusion proteins were prepared from E. coli and the quantity recovered was normalized based upon Coomassie-stained gel analysis. GST pull down was performed with cell lysate from cells that transiently expressed the HA-C-subunit (Fig. 5A). All of the constructs, including the non-overlapping d238 and 238Δ fusions, pulled down the PP2A C-subunit. To our surprise, 53 Δ, 155 Δ and 175 Δ showed stronger PP2A binding than the full Na^+^,K^+^-loop or 238 Δ and 272 Δ. These data suggest the possibility there is a region between amino acids 1 and 175 in the Na^+^,K^+^-loop that inhibits PP2A binding. Alternatively, the conformation of the Na^+^,K^+^-loop may regulate its association with PP2A and certain deletions may not conserve the requisite conformation.

### Association of the PP2A-A subunit with the Na^+^,K^+^-ATPase

The PP2A A-subunit was not pulled down with the large cytoplasmic loop of the Na^+^,K^+^-ATPase (Fig. 4) whereas the A-subunit was immunoprecipitated with the full length Na^+^,K^+^-ATPase (Fig. 2 and 3). It is possible that there is another site in the Na^+^,K^+^-ATPase sequence that associates with PP2A A-subunit, since a number of PP2A substrates have been found to bind to PP2A through the A-subunit [39], [40], [41]. We used a GST fusion protein incorporating the A-domain [42] of the Na^+^,K^+^-ATPase α-subunit, which is composed of the N-terminus and a small cytoplasmic loop connecting transmembrane segments 2 and 3, to test its capacity to pull down the PP2A A-subunit (Fig. 5B). The A-domain includes the PKC phosphorylation site [6] and also may be phosphorylated by G-protein coupled receptor kinases [28]. We found that the A domain pulled down the PP2A A-subunit. Consistent with the data presented in Fig. 4C, the large cytoplasmic loop of the Na^+^,K^+^-ATPase did not precipitate the PP2A A-subunit.

### Effect of PP2A on interaction of arrestin 2 with the Na^+^,K^+^-ATPase

**Figure 6:**
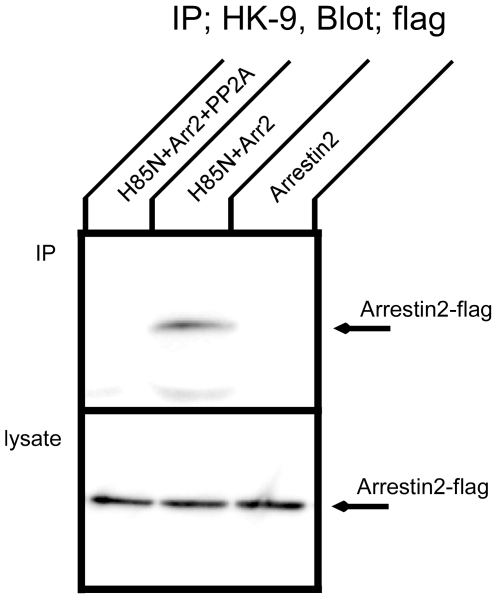
Co-immunoprecipitation of arrestin with the Na^+^,K^+^-ATPase in the presence of PP2A. COS cells were transfected with flag tagged arrestin 2 alone or H85N plus flag tagged arrestin 2 in the presence or absence of HA tagged PP2A C-subunit. Immunoprecipitation was performed with HK9 antibody directed against the N terminus of H85N, and arrestin 2 was detected by Western blotting with anti-flag antibody. Arrestin co-precipitated with the Na^+^,K^+^-ATPase α-subunit, and this association was partially inhibited in the presence of PP2A. Typical results from one of three experiments are shown.

Arrestin and GRK are major regulators of GPCR trafficking and signaling. We have found that Na^+^,K^+^-ATPase trafficking is also regulated by arrestin and GRK, together with spinophilin [28]. Since the association between arrestin and GPCRs depends upon the phosphorylation of GPCRs by GRKs, PP2A might conceivably regulate Na^+^,K^+^-ATPase function, at least in part, through inhibition of GRK phosphorylation and arrestin binding. To begin to test this hypothesis, we examined the effect of the PP2A C-subunit on the association of the Na^+^,K^+^-ATPase with arrestin (Fig. 6). Fig. 6 shows Western blot patterns of transfected COS cell lysates subjected to immunoprecipitation with the HK9 antibody and then detected with the anti-flag antibody, which recognizes arrestin 2. Arrestin 2 was co-immunoprecipitated by the HK9 antibody when it was co-expressed with H85N. Co-expression of the PP2A C-subunit completely inhibited the interaction between arrestin 2 and the H85N α-subunit. Thus, the PP2A C-subunit appears to impede arrestin binding to the Na,K-ATPase α-subunit. This effect could be attributable to the catalytic activity of the PP2A C-subunit, through the dephosphorylation of phosphoresidues that may be important for the arrestin interaction. Alternatively, the inhibitory influence of the PP2A subunit on arrestin binding to the Na,K-ATPase may be simply due to steric competition between these two polypeptides for the same or overlapping binding sites on the α-subunit. To assess this possibility, we performed a competitive binding experiment.

### Binding competition between arrestin and PP2A C-subunit

**Figure 7:**
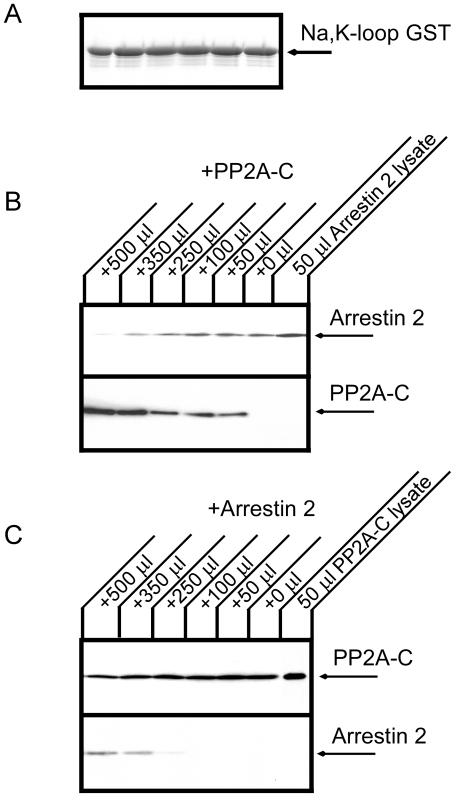
Competition between PP2A and arrestin for binding to the large cytoplasmic loop of the Na^+^,K^+^-ATPase. COS cell lysates expressing flag tagged arrestin 2 or HA tagged PP2A C-subunit were prepared. A. Coomassie Brilliant Blue staining demonstrating that the same quantities of GST fusion protein incorporating the large cytoplasmic loop of the Na^+^,K^+^-ATPase were used in each lane of the experiments depicted in panels B and C. B. GST fusion protein incorporating the large cytoplasmic loop of the Na^+^,K^+^-ATPase was incubated with 500 µl of lysate from arrestin2-expressing cells and varying amounts of lysate from PP2A C-subunit-expressing cells. Arrestin 2 (upper panel) and PP2A C-subunit (lower panel) were detected by western blotting using an anti-flag and anti-HA antibodies, respectively. C. GST fusion protein incorporating the large cytoplasmic loop of the Na^+^,K^+^-ATPase was incubated with 500 µl of lysate from PP2A C-subunit-expressing cells and varying amounts of lysate from arrestin 2-expressing cells. PP2A C-subunit (upper panel) and arrestin 2 (lower panel) were detected by western blotting with an anti-HA and anti-flag antibodies, respectively. Arrestin 2 binding to the Na^+^,K^+^-ATPase large loop was strongly inhibited by the PP2A C-subunit. Typical results from one of three experiments are shown.

As shown in Fig. 6, the PP2A C-subunit partially disrupted the association between the Na^+^,K^+^-ATPase and arrestin 2. Since both arrestin 2 and PP2A C-subunit interact with the Na^+^,K^+^-ATPase large cytoplasmic loop, the PP2A C-subunit may directly block arrestin binding to the large cytoplasmic loop of the Na^+^,K^+^-ATPase. Fig. 7B shows a pull down experiment testing the association between a GST protein incorporating the large cytoplasmic loop of the Na^+^,K^+^-ATPase and arrestin 2 in the presence of PP2A C-subunit. PP2A C-subunit strongly inhibited arrestin binding to the large cytoplasmic loop of the Na^+^,K^+^-ATPase. Fig. 7C shows the converse experiment, in which the interaction between the GST protein incorporating the large cytoplasmic loop of the Na^+^,K^+^-ATPase and PP2A C-subunit was tested in the presence of arrestin 2. In contrast to the results presented in Fig. 7B, PP2A C-subunit binding to the Na^+^,K^+^-ATPase was only minimally inhibited in the presence of arrestin 2. Coomassie Brilliant Blue staining confirmed that equal amounts of GST protein were used in all lanes (Fig. 7A). These data suggest the interesting possibility that the affinity of PP2A C-subunit for binding to the sodium pump large cytoplasmic loop fusion protein is substantially higher than that of arrestin.

### Localization of the Na^+^,K^+^-ATPase in COS cells expressing arrestin and PP2A

**Figure 8:**
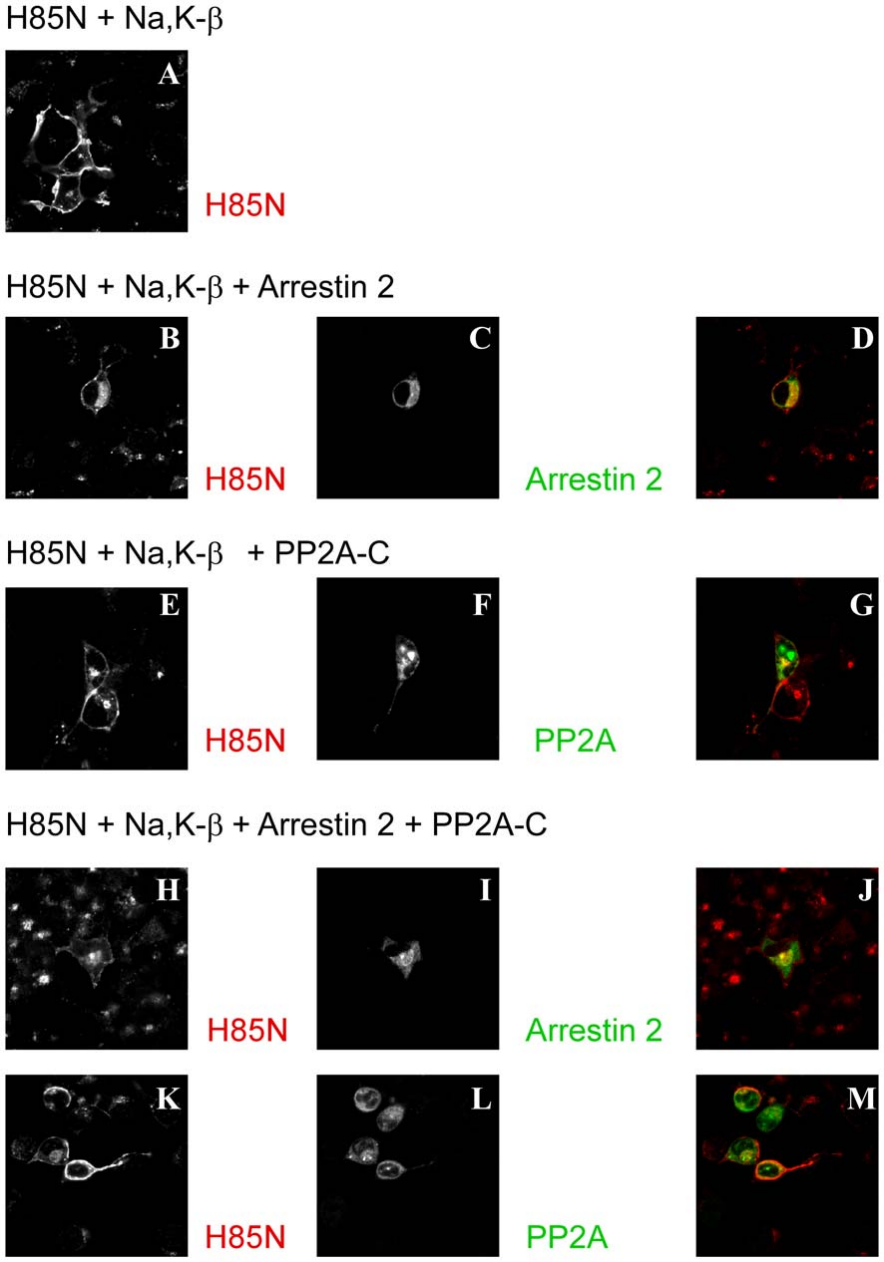
Immunolocalization of the Na^+^,K^+^-ATPase and arrestin in the presence of PP2A in COS cells. COS cells were transfected with H85N plus Na^+^,K^+^-ATPase β-subunit (A), with H85N, Na^+^,K^+^-ATPase β-subunit and flag tagged arrestin (B-D), with H85N, Na^+^,K^+^-ATPase β-subunit and HA tagged PP2A C-subunit (E-G), or with H85N plus Na^+^,K^+^-ATPase β-subunit and flag tagged arrestin plus HA tagged PP2A C-subunit (H-M). Cells were stained with HK9 (A, B, E, H and K), anti-flag for arrestin (C and I), and anti-HA for PP2A C-subunit (F and L) antibodies. Overlay patterns are shown in D, G, J and M. (×200 magnification) A large fraction of the H85N was found in intracellular compartments when cells expressed arrestin in the absence of PP2A C-subunit. This effect was not observed when arrestin was expressed together with the PP2A C-subunit. Typical results from one of three experiments are shown.

We have shown that arrestin over-expression induces the redistribution of the Na^+^,K^+^-ATPase to intracellular compartments [28]. Since the PP2A C-subunit inhibited arrestin binding (Fig. 6 and 7), we investigated the effect of the PP2A C-subunit on the localization of the Na^+^,K^+^-ATPase co-expressed with arrestin (Fig. 8). COS cells were transfected with H85N plus Na^+^,K^+^-ATPase β-subunit in the presence or absence of arrestin 2 and/or PP2A C-subunit and cells were stained with flag antibody to detect arrestin 2 (C and I), with the HK9 antibody to determine the distribution of the H85N (A, B, E, H and K) and with HA antibody to detect the PP2A C-subunit (F and L). Arrestin 2 was expressed in association with cytoplasmic structures either in the absence or in the presence of PP2A (C, D, I and J). When cells were transfected with arrestin 2 in the absence of PP2A C-subunit, a substantial fraction of the H85N was also localized intracellulary (B) and appeared to co-localize with arrestin 2 (D). With over-expression of PP2A C-subunit, however, the H85N was not co-localized with arrestin 2 and instead was found predominantly at the cell surface (H, K, J and M). The PP2A C-subunit itself exerted no apparent effect on the localization of the Na^+^,K^+^-ATPase in the absence of arrestin (E and G). These results indicate that PP2A regulates the effects of arrestin on Na^+^,K^+^-ATPase localization.

### In vitro phosphorylation of the large cytoplasmic loop of the Na^+^,K^+^-ATPase by GRKs in the presence and absence of PP2A

**Figure 9:**
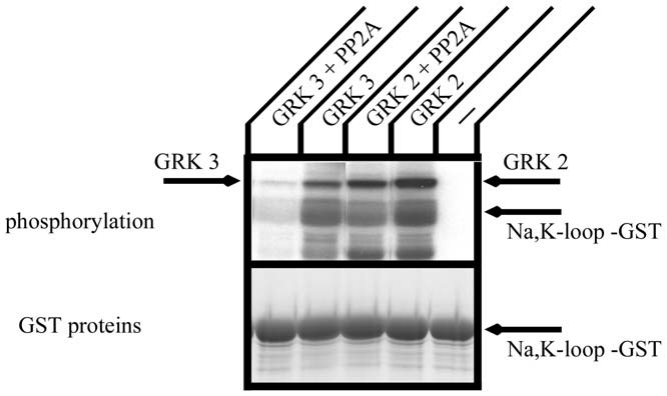
In vitro phosphorylation of the large cytoplasmic loop of the Na^+^,K^+^-ATPase α-subunit. A. GST construct incorporating the large cytoplasmic loop of the Na^+^,K^+^-ATPase α-subunit was phosphorylated with [γ-^32^P]-ATP by GRKs prepared by immunoprecipitation from COS cell lysates. Phosphorylation was performed in the presence or absence of PP2A at 22°C for 30 min. Reactions were stopped by adding SDS-PAGE sample buffer and samples were separated by SDS-PAGE. The gel was stained with Coomassie Brilliant Blue, dried, and analyzed by autoradiography (upper panel). The Coomassie Brilliant Blue staining demonstrating the expression levels of the GST proteins that were used for in vitro phosphorylation is shown in the lower panel. GRK 2 and GRK 3 phosphorylated the large cytoplasmic loop α-subunit, and PP2A partially reversed this phosphorylation. Typical results from one of four experiments are shown.

We have shown that the Na^+^,K^+^-ATPase associates with GRKs, which phosphorylate its large cytoplasmic loop [28]. As PP2A is one of the major phosphatases in cells, phosphorylation of the Na^+^,K^+^-ATPase by GRKs may be regulated by PP2A. We tested the possibility that GRK phosphorylation of the Na^+^,K^+^-ATPase is regulated by PP2A (Fig. 9). GRK 2 and 3 were prepared by immunoprecipitation from lysates obtained from COS cells transfected with GRK. Neither GRK 2 nor GRK 3 phosphorylated GST alone [28] . Both GRK 2 and GRK 3 phosphorylated the large cytoplasmic loop of the Na^+^,K^+^-ATPase (Fig. 9). PP2A partially inhibited phosphorylation by GRK 2 and completely eliminated phosphorylation of the large cytoplasmic loop of the Na^+^,K^+^-ATPase by GRK 3. PP2A also completely eliminated the detection of GRK 3 auto phosphorylation activity. These results indicate that PP2A has the potential to control GRK phosphorylation of the Na^+^,K^+^-ATPase.

## Discussion

We have found that PP2A interacts with the Na^+^,K^+^-ATPase [17]. This interaction appears to be involved in the regulation of the Na^+^,K^+^-ATPase, since it has been shown that the activity of the Na^+^,K^+^-ATPase is governed by phosphorylation and dephosphorylation through the action of kinases and phosphatases [6], [29], [30], [31], [32], [33], [34], [35]. Here we show that the Na^+^,K^+^-ATPase directly binds to PP2A. Furthermore, this binding leads to at least partial dephosphorylation of the Na^+^,K^+^-ATPase α-subunit at its GRK phosphorylation sites. We also demonstrate that the expression of PP2A reduces the interaction between the Na^+^,K^+^-ATPase and arrestin, and abolishes arrestin's effect on pump trafficking.

Several putative PP2A binding sequence with other proteins such as the ryanodine receptor [43], [44] and janus kinase 2 [45] were reported, however, such canonical PP2A binding sequences do not appear to be represented in the primary structure of the Na^+^,K^+^-ATPase α-subunit. We have begun to map the interaction site for PP2A in the large cytoplasmic loop of the Na^+^,K^+^-ATPase α-subunit using GST pull down assays. Our results show that both ends of the large cytoplasmic loop of the Na^+^,K^+^-ATPase α-subunit associate with the PP2A C-subunit. Moreover, PP2A A-subunit associates with the A-domain of the Na^+^,K^+^-ATPase α-subunit. These results show that the Na^+^,K^+^-ATPase has at least three potential sites for binding to the PP2A holoenzyme. Multiple sites for interaction with PP2A may be necessary to permit PP2A to participate in the regulation of several widely spaced phosphorylation sites. For example, the PKA phosphorylation site, which is dephosphorylated by PP2A, resides at the C-terminal end of the Na^+^,K^+^-ATPase α-subunit [46], [47], [48] and the PKC phosphorylation site is located in the A-domain of the Na^+^,K^+^-ATPase α-subunit [47], [49], [50]. It has been shown that catalytic PP2A C-subunit directly associates with the first 90 amino acids of the Na^+^,K^+^-ATPase α-subunit and dephosphorylates the Na^+^,K^+^-ATPase at PKC site [18]. We have also shown that the A-domain and the large cytoplasmic loop of the Na^+^,K^+^-ATPase are susceptible to phosphorylation by GRKs [28]. We suggest, therefore, that it is possible that PP2A can associate with several pump domains close to these phosphorylation sites and thus mediates the regulated dephosphorylation of the Na^+^,K^+^-ATPase α-subunit.

PP2A appears to serve this function for the class C L-type calcium channels [51] and for neurotransmitter transport proteins [52]. Class C L-type calcium channels (Ca_v_1.2) are subject to PKA phosphorylation following β-adrenergic stimulation, which increases their channel activity [53]. Furthermore, the phosphatase inhibitor okadaic acid increases the PKA-stimulated activity of class C channels [54]. Recent studies showed that the PP2A C-subunit directly and stably associates with this channel. This interaction did not depend on the phosphorylation state of the channel and was able to regulate channel activity [51]. On the other hand, different effects have been documented for neurotransmitter transport proteins [52]. Dopamine, norepinephrine, and serotonin transporters are rapidly regulated by direct or receptor mediated activation of cellular kinases, particularly PKC. The PP2A C-subunit associates with all of these transporters. PKC activation leads to disassociation of the transporter/PP2A complexes, and this effect can be blocked by the transporter's substrate.

We have tested whether PKA or PKC activation changes the sodium pump's association with PP2A using the H85N α-subunit construct and HA tagged PP2A C-subunit expressed in COS cells. Both PKC and PKA activation by PMA and forskolin plus IBMX, respectively, had no effect on the extent of the co-immunoprecipitation of the Na^+^,K^+^-ATPase with the PP2A C-subunit (data not shown). Thus, the PP2A C-subunit and the Na^+^,K^+^-ATPase may associate stably with PP2A, and phosphorylation of the Na^+^,K^+^-ATPase α-subunit may not affect this interaction, as is the case for the class C L-type calcium channels. It should also be noted that since the Na^+^,K^+^-ATPase appears to possess multiple PP2A binding sites, it is possible that each of these binding sites might show different individual affinities and phosphorylation dependence for PP2A binding.

Renal Na^+^,K^+^-ATPase activity is regulated by several hormones, including dopamine, β-adrenergic hormone and arginine vasopressin [55], [56], [57], [58]. Evidence suggests that this regulation occurs through phosphorylation and de-phosphorylation of the Na^+^,K^+^-ATPase α-subunit. Phosphorylation of the Na^+^,K^+^-ATPase α-subunit regulates not only its activity, but also its intracellular trafficking. We propose a new mechanism for regulation of the trafficking and activity of the Na^+^,K^+^-ATPase by hormones though the action of GRKs, arrestins and PP2A. β-adrenergic receptor kinases (βARKs) were initially described and named as an activity that phosphorylated the agonist-occupied β_2_-adrenergic receptor, but not inactive receptor [59]. The form of the kinase that was originally purified and cloned is now called GRK2 (βARK1) [60] and a highly related isoform, GRK3 (βARK2) [61], has been identified. The substrate specificity of these GRKs is not limited to GPCRs, and a recent study shows that the β-subunit of the epithelial Na^+^ channel is phosphorylated and regulated by GRK 2 [62]. It has been shown that the activity of the epithelial Na^+^ channel (ENaC) in the distal nephron is regulated by the GPCRs that respond to antidiuretic hormone and vasopressin [63] . Dinudom et al have shown that GRK 2, as well as PKA and PKC, phosphorylate and increase activity of EnaC, while protein phosphatase 1 (PP1) inhibits this effect through de-phosphorylation of ENaC [62]. GRK-catalyzed phosphorylation of GPCRs is essential for initiating their binding with arrestins. Arrestin 2 and 3 (also called β-arrestin 1 and 2, respectively) interact almost exclusively with specific phospho-serine/threonine residues of ligand-activated seven membrane spanning GPCRs [64], [65]. A recent study shows that the Na^+^/H^+^ exchanger isoform NHE5 also associates with arrestin 2 and 3, and that this association leads to decreased cell surface abundance of this transporter [66]. It has been shown that the NHE3 Na^+^/H^+^ exchanger isoform, which shares a great deal of sequence identity with NHE5, is regulated by a number of GPCRs. It is tempting to suggest that transporters such as the Na^+^,K^+^-ATPase, whose activities are controlled by GPCRs, interact with GRKs and arrestins, and that protein phosphatases such as PP2A regulate these transporters at least in part through effecting the reversal of the effects induced by GRK mediated phosphorylation.

In summary, we have shown that PP2A associates directly with Na^+^,K^+^-ATPase and not with the gastric H^+^,K^+^-ATPase. The PP2A C-subunit is necessary for association of PP2A with the large cytoplasmic loop of the Na^+^,K^+^-ATPase and the PP2A A-subunit is able to associate at the A-domain of the Na^+^,K^+^-ATPase α-subunit. PP2A inhibits GRK phosphorylation of the pump's large cytoplasmic loop. Future studies will be necessary to determine the role of this novel interaction in regulating the involvement of the Na^+^,K^+^-ATPase in a variety of physiologically important processes.

## Materials and Methods

### Antibodies

Anti-Na^+^,K^+^-ATPase monoclonal antibody, 6H is directed against the amino terminus of the Na^+^,K^+^-ATPase α-subunit [67]. Anti-H^+^,K^+^-ATPase polyclonal antibody HK9 is directed against the amino terminus of the H^+^,K^+^-ATPase α-subunit [68]. Anti-PP2A A-subunit and C-subunit antibodies were purchased from Upstate (Charlottesville, VA). Anti-HA antibody was obtained from Covance (Berkeley, CA), anti-flag antibody was obtained from Sigma, and anti-Xpress antibody was purchased from Invitrogen (Carlsbad, CA).

### Biotinylation of Anti-Na^+^,K^+^-ATPase monoclonal antibody, 6H

6H was dialyzed against PBS at 4°C, diluted to 1 mg/ml with PBS, and NaHCO_3_ was added to 50 mM, final concentration. EZ-Link Sulfo-NHS SS-Biotin (PIECE, Rockford, IL) was mixed at 0.25 mg Biotin/1 mg 6H and samples were incubated overnight. Excess biotin was removed by dialysis against PBS. Biotinylated 6H had almost the same reactivity by Western blotting as non-biotinylated 6H (data not shown).

### Plasmid construction

The A domain of the rat Na,K-ATPase α-subunit was amplified by polymerase chain reaction [17]. This construct was subcloned as a *BamHI/EcoRI* fragment into the pGEX-4T-3 vector (Amersham-Pharmacia, Piscataway, NJ) to produce a cDNA encoding a GST-fusion protein. The large cytoplasmic loop connecting the TM4+TM5 of the Na^+^,K^+^-ATPase α-subunit was amplified by PCR with primers that included EcoR I and Not I restriction sites. The PCR fragment was subcloned into pGEX-4T-3 vector, in which the insert was fused to the carboxyl terminus of glutathione S-transferase. To generate deletions, BspEI, ClaI, MfeI and HindIII sites were introduced in the pGEX-4T3 construct by creating silent mutations. Mutated constructs were digested with NotI plus BspEI, NarI, ClaI, MfeI or HindIII for C-terminal deletions or EcoRI and ClaI for the N-terminal deletion. Small fragments were removed by agarose gel electrophoresis, blunt ends were introduced with pfu DNA polymerase and the modified constructs were recircularized by ligation. The H85N chimera, which is composed of the H^+^,K^+^-ATPase (from amino acid 1 to 85) and the Na^+^,K^+^-ATPase (from amino acid 86 to the C-terminus, was subcloned into the mammalian expression vector pCB6 as previously described [38]. PP2A C-subunit was cloned by PCR from a human kidney cDNA library. PCR was performed with primers that included Kpn I and Xba I restriction sites, and sequence encoding an HA epitope tag. The HA tag was fused to the amino terminus of the resulting construct. The PCR fragment was subcloned into the mammalian expression vector pcDNA 3.1(+). PP2A A-subunit was also cloned by PCR with primers that included Kpn I and Xba I restriction sites, and sequence encoding a flag epitope tag. The PCR fragment was subcloned into the pcDNA 3.1(+) vector. The flag epitope was fused to the amino terminus of the PP2A A-subunit construct. All PCR primer sequences are available on request. Arrestin 2 and 3, and GRK 2 and 3 were cloned by PCR from a human kidney cDNA library. PCR was performed with primers that included Kpn I and Xba I restriction sites, and sequence encoding a flag or HA epitope tags. The flag and HA tags were fused to the amino terminus of the resulting constructs for arrestin 2 and 3, and to the carboxyl terminus for GRK 2 and 3. The PCR fragments were subcloned into the mammalian expression vector pcDNA 3.1(+).

### Cell culture and transfection

COS cells were cultured in a humidified incubator under 5% CO_2_ in α-MEM (GIBCO, Carlsbad, CA) supplemented with 10% FBS, 2 mM L-glutamine, 50 U/mL penicillin and 50 µg/ml streptomycin. DNA transfection was performed with Lipofectamine 2000 (Invitrogen, Carlsbad, CA) according to the manufacturer's instructions, and assays were performed 48 h after transfection.

### Immunoprecipitation

Transfected cells were incubated with 1 ml of lysis buffer containing 1% Triton X-100, 150 mM NaCl, 5 mM EDTA, and 25 mM Tris-HCl, pH 7.4 for 30 min at 4°C. Insoluble material was removed through centrifugation at 10,000 g for 30 min at 4°C. After centrifugation, 20 µl of lysate was saved to assess the level of expression of the transfected constructs. The rest of the lysate was incubated overnight at 4°C with the specific antibody of interest and protein -A or -G agarose beads (Pierce, Rockford, IL). The bead complexes were washed 4 times with washing buffer containing 0.1% NP-40, 0.1% Tween 20, 500 mM NaCl and 10 mM Tris-HCl, pH8.0 and once with PBS. Proteins were eluted in SDS-PAGE sample buffer. The samples were separated by SDS-PAGE and analyzed by Western blotting.

### Immunohistochemistry

ICR mice were anesthetized and the internal organs were fixed as described by Biemesderfer et al. [69]. The kidneys were cut at 2 µm thickness on a Microme HM500M cryostat. Tissue was incubated with anti-PP2A polyclonal antibody (Cell Signaling, MA) and anti-Na^+^,K^+^-ATPase monoclonal antibody, α5, followed by anti-mouse Alexa Fluor 488 and anti-rabbit Alexa Fluor 568 conjugated IgG (Molecular Probes, Eugene, CR). Fluorescence was visualized with an Olympus Fluoview FV500 laser confocal microscope. Images are the product of 4-fold line averaging. Contrast and brightness settings were chosen so that all pixels were within the linear range. All animal experiments were conducted in accordance with the policies and procedures of the Yale IACUC (permit number 07267) and The Experimental Animal Ethics Committee in Kyorin University (permit number 92-1).

### Rat tissue preparation and immunoprecipitation

Sprague-Dawley rat kidneys were removed under anesthesia and washed with cold PBS. The kidneys were minced in lysis buffer containing 4% CHAPS, 150 mM NaCl, 5 mM MgCl_2_, and 25 mM HEPES, pH 7.4. The minced kidneys were sonicated, homogenized and sonicated again. The insoluble fraction was removed through two successive centrifugations at 18,000×g for 30 min at 4°C. Supernatant was incubated with PP2A A- or C-subunit antibody overnight and protein A beads were added for 5 hrs. The beads were washed with lysis buffer 4 times, followed by washing with PBS. Proteins were separated by SDS-PAGE and Western blot analysis was performed with biotinylated anti- Na^+^,K^+^-ATPase α-subunit antibody (6H) and streptavidin HRP secondary (Zymed, South San Francisco, CA). Specific antibody binding was detected by ECL (Amersham-Pharmacia, Piscataway, NJ).

### In vitro transcription/translation and GST pull down assay

In vitro translation was performed with the TNT coupled reticulocyte lysate system (Promega, Madisow, WI) according to the product manual. HA-tagged PP2A C-subunit or Xpress tagged PP2A A-subunit in pcDNA3.1, which has a T7 promoter, was used as a template. A pGEX construct including the large cytoplasmic loop of the Na^+^,K^+^-ATPase α-subunit was transformed into E.coli BL-21. The expression of GST fusion protein was induced with 0.1 mM IPTG and a protein extract was prepared with 1% Triton X-100 in PBS. The extract was incubated with Glutathione Sepharose™ 4B beads (Amersham-Pharmacia, Piscataway, NJ) for 6 hrs at 4°C. Non-specific binding was blocked with 0.1% BSA in PBS for 1 hr and beads were incubated with translated products. After incubation, these beads were washed 4 times with washing buffer containing 1% Tween 20, 1% NP-40, 500 mM NaCl and 10 mM Tris-HCl, pH8, and 1 time with PBS. Specifically adherent polypeptides were eluted in SDS-PAGE sample buffer and analyzed by SDS-PAGE and Western blotting.
